# Phylogenetic analysis of HIV-1 archived DNA in blood and gut-associated lymphoid tissue in two patients under antiretroviral therapy

**DOI:** 10.1186/s13099-021-00416-6

**Published:** 2021-03-23

**Authors:** Patricia Recordon-Pinson, Annie Gosselin, Petronela Ancuta, Jean-Pierre Routy, Hervé Fleury

**Affiliations:** 1 CNRS UMR 5234, Université de Bordeaux, Bordeaux, France; 2 Centre Hospitalier Universitaire de Montreal (CHUM) Research Centre, Montréal, QC Canada; 3 Département de Microbiologie, Infectiologie Et Immunologie, Faculté de Médecine, Université de Montréal, Montréal, QC Canada; 4 Chronic Viral Illness Service and Division of Hematology, McGill University Health Centre, Montreal, QC Canada; 5 CHU de Bordeaux, Bordeaux, France

**Keywords:** HIV-1, Proviral DNA, Blood, Gut associated lymphoid tissue (GALT), Phylogenetic comparison of archived viral sequences

## Abstract

One of the approaches to cure human immunodeficiency virus (HIV) is the use of therapeutic vaccination. We have launched the Provir/Latitude 45 study to identify conserved CTL epitopes in archived HIV-1 DNA according to the HLA class I alleles in aviremic patients under antiretroviral therapy (ART). A HIV-1 polypeptidic therapeutic vaccine based on viral sequence data obtained from circulating blood was proposed; here, our aim was to compare the proviral DNA in blood and gut-associated lymphoid tissue (GALT). Peripheral blood mononuclear cells and gut biopsies were obtained from two HIV-1 infected patients under successful antiretroviral therapy. Total DNA was extracted including the proviral DNA. The HIV-1 reverse transcriptase was sequenced in both compartments using next generation sequencing followed by single genome sequencing; phylogenetic trees were established and compared. The proviral sequences of both compartments intra-patient exhibited a very low genetic divergence while it was possible to differentiate the sequences inter-patients; single genome sequencing analysis of two couples of samples confirmed that there was no compartmentalization of the sequences intra-patient. We conclude that, considering these two cases, the proviral DNA sequences in blood and GALT are similar and that the epitope analysis of HIV-1 provirus in blood should be considered as relevant to that observed in the GALT, a hard-to-reach major compartment, and can therefore be used for therapeutic vaccine approaches.

## Background

HIV-1 infection can be managed by ART, leading to the control of viral replication and improving the health of people living with HIV. However, ART cannot be interrupted since this would lead to a rebound of viral replication [1, 2] as virus establishes cellular (latently infected resting CD4 + memory T cells) and anatomical reservoirs very early during infection [3–8]. Gut Associated Lymphoid Tissue (GALT) is considered to be one of the main reservoirs of Simian Immunodeficiency Virus (SIV) and HIV infection [9–11]. Cure strategies for HIV-1 include therapeutic vaccination [12], although immune response observed was not able to control viral replication after ART discontinuation [13]. In this context, we launched the Provir/Latitude 45 project to identify conserved CTL epitopes in the proviral HIV-1 DNA of patients with long-term ART [14]. The study involves in silico modeling based on the HIV-1 proviral DNA sequences, the HLA alleles and the HIV-1 CTL epitopes following sequencing of the archived DNA from peripheral blood mononuclear cells (PBMC), i.e., from circulating blood. Since our initial work was based on proviral DNA in PBMC, we assessed whether our observations would be the same in another compartment, namely GALT. Herein, we present a phylogenetic comparison of the archived HIV-1 DNA in PBMC and GALT from two HIV-1 infected patients at success of ART.

## Materials and methods

The study participants (referenced as patients 5A and 10) were recruited at Centre Hospitalier Universitaire de Montreal (CHUM). They were Caucasian individuals infected with an HIV-1 subtype B and under successful antiretroviral therapy (ART) for more than 4 years. For each patient, a sigmoid biopsy was collected during colonoscopy 4 years after initiation of successful ART and processed as previously described [15]. Matched peripheral blood was collected on the same day and immediately processed with Ficoll for PBMC isolation, performed in parallel with cell extraction from biopsy tissue. DNA was extracted from both compartments and used for next generation sequencing (NGS) analysis of HIV-1 provirus; we used the method already published [16, 17] to amplify fragment B, i.e., polymerase (Pol) region including RT and integrase. The PCR products were purified and quantitated, the library was prepared using the Nextera XT DNA Sample Preparation kit; each individual library was then sequenced on a MiSeq Illumina platform. Raw data (FASTQ files) were submitted to the SmartGene^®^ NGS HIV-1 module to generate a BAM file for each patient and each sample was processed for further analysis [18]. The study was carried out using only the Pol RT part region of the sequences obtained. Using Galaxy and Clustal software, RT gene sequences from the two compartments (GALT and PBMC) were selected for neighbor-joining analysis from matrix distances calculated after gapstripping of alignments, with a Kimura two-parameter algorithm and bootstrap analysis. To do so, an alignment was generated that included only reads with lengths > 400 bp corresponding to a given region of RT (variable according to the patient, from amino-acids 39 to 202). This length limitation explains the small number of reads used for this analysis compared to the total number of reads covering this region. Phylogenetic trees were visualized using Interactive Tree of Life (ITOL) software [19]. Single genome sequencing (SGS) was carried out according to the method of Palmer et al. [20]. The total extracted DNA of both compartments was diluted in TE buffer at a dilution yielding a PCR product in three out of 10 PCRs. In this case, according to Poisson’s distribution, the dilution contains one copy of cDNA per positive PCR at about 80% of the time. Two rounds of PCR for RT amplification were followed by visualization of the PCR products. The 1:9 dilution was found to be optimal for Sanger sequencing and the sequences (assuming that there was no mixture of population) of PBMC and GALT obtained were aligned by Clustal to obtain a neighbor-joining tree. For evaluation of evolutionary divergence, the median, mean and range of the number of base substitutions per site between RT sequences were calculated. Analyses were conducted using the Maximum Composite Likelihood model [21]. The analysis involved 4100 nucleotide sequences. Codon positions included were 1st + 2nd + 3rd + Noncoding. All positions containing gaps and missing data were eliminated. There was a total of 376 positions in the final dataset. Evolutionary analyses were conducted in Molecular Evolutionary Genetics Analysis (MEGA) 7 software [22].

## Results

**Fig. 1:**
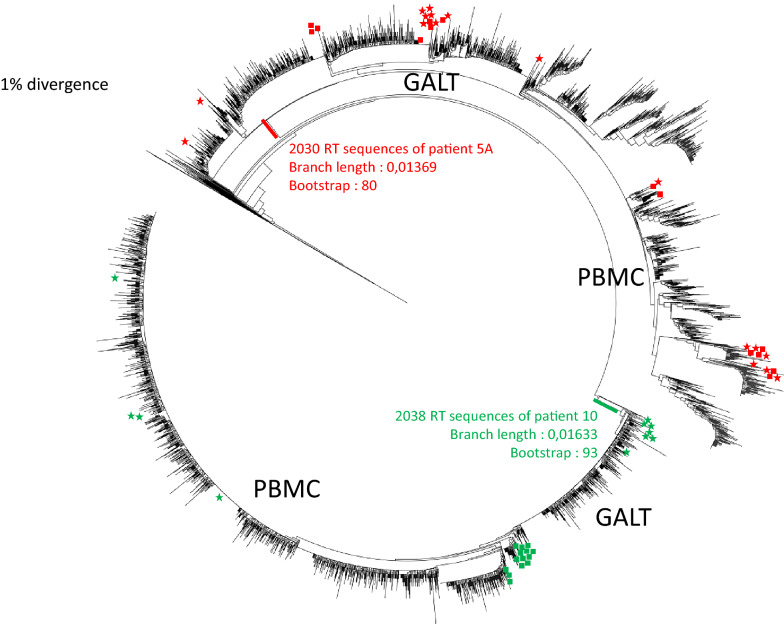
Phylogeny of RT sequences of PBMC and GALT from patients 5A and 10. The NGS sequences are in black. SGS: the symbols denote sampling location and patients: patient 5A PBMC (red star), patient 5A GALT (red square), patient 10 PBMC (green star), patient 10 GALT (green square)

The phylogenetic trees are presented in Fig. 1.

NGS analysis of patient 5A shows that the sequences from blood and GALT compartments exhibit a low genetic divergence and are located on the same branch; however, there is a slight divergence between these sequences and the SGS analysis allows to demonstrate that there is a true intermingling of the sequences, therefore evidencing a lack of compartmentalization.

NGS analysis of patient 10 first indicates, compared to the NGS data from patient 5A, that we can fully differentiate the HIV-1 isolates from both patients although they are of the same subtype (B subtype); focusing again on patient 10, we can draw the same conclusion, as for patient 5A, on the low genetic divergence of the sequences between both compartments; when analysing the SGS data, the clonal PBMC sequences are located at the origin of the GALT NGS part of the tree, then found in the PBMC part of the NGS tree while GALT clonal sequences are located at the end of the GALT tree and the origin of the PBMC tree; as for patient 5A we can conclude that there is no evidence of compartmentalization.

To confirm that all the sequences were clustered by patient, we estimated the evolutionary divergence between sequences (Table 1) considering patients 5A and 10. HIV-1 clusters were identified at maximum genetic distances between 4.5 and 7.5% and bootstrap support threshold varied between 70 and 99% [23]. As sequences from patients 5A and 10 are assembled with a median divergence of 5.3% and 2.2% respectively with bootstrap values of 80% and 93%, we confirm that these sequences from GALT and PBMC formed a specific cluster per patient.

**Table 1:** Estimates of evolutionary divergence between patients’ HIV-1 proviral DNA sequences

	Patient 5A		Patient 10		Patients 5A + 10	
	Median [min–max]	Mean	Median [min–max]	Mean	Median [min–max]	Mean
GALT						
NGS	0.030 [0–0.189]	0.029	0.016 [0–0.098]	0.016	nd	nd
SGS	0.055 [0–0.088]	0.047	0.005 [0–0.013]	0.005	nd	nd
NGS + SGS	0.061 [0–0.119]	0.054	0.011 [0–0.065]	0.012	nd	nd
PBMC						
NGS	0.053 [0–0.145]	0.051	0.013 [0–0.061]	0.013	nd	nd
SGS	0.047 [0–0.122]	0.051	0.013 [0–0.030]	0.013	nd	nd
NGS + SGS	0.065 [0.013–0,132]	0.065	0.016 [0–0.057]	0.017	nd	nd
GALT + PBMC						
NGS	0.053 [0.008–0.143]	0.054	0.022 [0.003–0.095]	0.022	0.08 [0.047–0.214]	0.085

Evolutionary divergences are based on the number of base substitutions per site among RT NGS and/or SGS sequences obtained from GALT and/or PBMC proviral DNA of patients 5A and 10

*nd* not done, *RT* reverse transcriptase, *GALT* gut associated lymphoid tissue, *PBMC* peripheral blood mononuclear cells, *NGS* next generation sequencing, *SGS* single genome sequencing

## Discussion

Archived viral DNA is found in intestinal tissue at a higher concentration than in PBMCs in ART patients [24], although the distribution of DNA in CD4 + CCR7 + , transitional memory and effector memory CD4 + T cells is different in blood and intestinal compartments [25]. GALT is therefore a compartment of major importance in the pathophysiology of HIV infection [26, 27]. The data presented here are not related to the quantification of archived DNA but rather to a comparison of the RT sequences obtained by NGS in GALT and PBMC. In that regard, Van Marle et al. [28] have studied biopsies from infected untreated individuals and sequenced the nef and RT genes of the viral RNA from blood (PBMC) and different parts of the gut by cloning and Sanger sequencing; they concluded that there is a compartmentalization of the virus in the gut reservoir. On the other hand, Lerner et al. [29] found a low diversity of the GALT and PBMC viruses in patients having experienced a voluntary treatment interruption while Imamichi et al. [30] did not demonstrate any difference between RNA and DNA sequences from gut and blood of patients chronically infected with HIV-1. Studying HIV-1 infected patients at early and chronic infection stages, Rozera et al. [31] found a more pronounced compartmentalization of proviral quasispecies in gut compared with PBMC samples in patients with early infection compared with chronic patients. The loss of gut/PBMC compartmentalization in more advanced stages of HIV infection was confirmed by longitudinal observation.

Regarding ART treated patients, Evering et al. [32] have studied the variability of the proviral DNA in the gut and blood compartments by SGS of the env part of the virus. They showed absence of evolution of the env sequences in the GALT and in PBMC; the authors mention that they cannot rule out the possibility of evolution in other viral genomic regions of HIV-1 such as pol which have not been investigated. Josefsson et al. [33] have compared the HIV DNA in PBMC and GALT from patients being on successful ART; they have used SGS technology and showed that there is no significant difference between the sequences from both compartments. They concluded that the HIV reservoir is stable on long-term suppressive ART and raise the hypothesis that the population of infected cells exhibiting a low variability of the virus could be maintained by homeostatic cell proliferation.

The patients of our study are similar to those of the two publications mentioned above i.e., ART- treated patients with controlled viral load and therefore a stable viral reservoir; the phylogenetic inferences obtained after NGS evidenced a very low genetic distance between the GALT and PBMC compartments intra-patient. On the other hand, it is possible to differentiate the GALT/PBMC sequences inter-patients; the SGS analysis performed plus the genetic divergence values after NGS and SGS are concordant with a high similarity between proviruses intra-patient. It must be underlined that the SGS technique decreases taq-induced recombination and nucleotide mis-incorporation, providing therefore a more reliable conclusion than conventional cloning [20].

Among the limitations of our study, we must note the fact that only the RT part of the proviral DNA has been considered and also that we have analyzed global archived DNA molecules without differentiating noninfectious and replication competent genomes [34]; however, more recent data show that even defective proviral DNA molecules can be expressed and yield viral proteins recognized by CTL T CD8 + lymphocytes [35].

In conclusion, our results confirm that the proviruses in GALT and PBMC are very similar in these patients under ART and who could be the target population of choice for a therapeutic vaccine and indicate that the analysis of the blood compartment can provide results that can extrapolated to the gut compartment, a major reservoir of HIV.

The strategies used in the future, whether they will be associated with a shock-and-kill approach (and a therapeutic vaccine is a part of this approach) or block-and-lock effect based on HIV silencing [36], will have to consider the proviral DNA of the archived virus not only in the blood but also in different tissue reservoirs including the gut.

## Data Availability

The NGS sequences are available in GenBank under accession number PRJN557560; the Sanger sequences of the SGS study are available in GenBank under accession numbers MN250226 to MN250287.

## Abbreviations

HIV: Human immunodeficiency virus
SIV: Simian immunodeficiency virus
ART: Antiretroviral therapy
GALT: Gut associated lymphoid tissue
NGS: Next generation sequencing
PBMC: Peripheral blood mononuclear cells
SGS: Single genome sequencing
Pol: Polymerase
RT: Reverse transcriptase
ART: Antiretroviral therapy
ITOL: Interactive tree of life
MEGA: Molecular evolutionary genetics analysis
